# Oleandrin, a cardiac glycoside, induces immunogenic cell death via the PERK/elF2α/ATF4/CHOP pathway in breast cancer

**DOI:** 10.1038/s41419-021-03605-y

**Published:** 2021-03-24

**Authors:** Xiaoxi Li, Jian Zheng, Shi Chen, Fan-dong Meng, Jing Ning, Shu-lan Sun

**Affiliations:** 1 Central Laboratory, Cancer Hospital of China Medical University, Liaoning Cancer Hospital & Institute, Shenyang, Liaoning 110042 People’s Republic of China; 2 Department of Thoracic Cancer, Cancer Hospital of China Medical University, Liaoning Cancer Hospital & Institute, Shenyang, Liaoning 110042 People’s Republic of China; 3 Molecular Oncology Laboratory of Cancer Research Institute, The First Affiliated Hospital of China Medical University, Shenyang, Liaoning 110001 People’s Republic of China; 4 Department of General Medicine (VIP ward) & Department of Tumor Supportive and Palliative Medicine, Cancer Hospital of China Medical University, Liaoning Cancer Hospital & Institute, Shenyang, Liaoning 110042 People’s Republic of China

**Keywords:** Tumour immunology, Preclinical research

## Abstract

Chemotherapeutic agents have been linked to immunogenic cell death (ICD) induction that is capable of augmenting anti-tumor immune surveillance. The cardiac glycoside oleandrin, which inhibits Na^+^/K^+^-ATPase pump (NKP), has been shown to suppress breast cancer growth via inducing apoptosis. In the present study, we showed that oleandrin treatment triggered breast cancer cell ICD by inducing calreticulin (CRT) exposure on cell surface and the release of high-mobility group protein B1 (HMGB1), heat shock protein 70/90 (HSP70/90), and adenosine triphosphate (ATP). The maturation and activation of dendritic cells (DCs) were increased by co-culturing with the oleandrin-treated cancer cells, which subsequently enhanced CD8^+^ T cell cytotoxicity. Murine breast cancer cell line EMT6 was engrafted into BALB/c mice, and tumor-bearing mice were administered with oleandrin intraperitoneally every day. Oleandrin inhibited tumor growth and increased tumor infiltrating lymphocytes including DCs and T cells. Furthermore, the differential mRNA expression incurred by oleandrin was investigated by mRNA sequencing and subsequently confirmed by quantitative real-time polymerase chain reaction (qRT-PCR) and western blotting. Mechanistically, oleandrin induced endoplasmic reticulum (ER) stress-associated, caspase-independent ICD mainly through PERK/elF2α/ATF4/CHOP pathway. Pharmacological and genetic inhibition of protein kinase R-like ER kinase (PERK) suppressed oleandrin-triggered ICD. Taken together, our findings showed that oleandrin triggered ER stress and induced ICD-mediated immune destruction of breast cancer cells. Oleandrin combined with immune checkpoint inhibitors might improve the efficacy of immunotherapy.

## Introduction

Breast cancer is the most common malignant tumor occurring in women. With the incidence and mortality rate of breast cancer increasing annually, this malignancy has remained as one of the most serious threats to women’s health. In 2019, over 2 million cases of breast cancer were reported worldwide, and more than half a million patient death was attributed to breast cancer^1^. Treatment options of breast cancer include surgery, radiotherapy, chemotherapy, endocrine therapy, and targeted therapy^2^. However, treatment outcome is still far from satisfactory, especially for triple-negative breast cancers that lack effective treatment targets.

In recent years, immunotherapies represented by immune checkpoint inhibitors have made remarkable achievements in tumor treatment. Phase III clinical trial has showed that paclitaxel combined with atezolizumab, a programmed death-ligand 1 (PD-L1)-blocking antibody, has significantly prolonged the progression-free survival and total survival of participants with metastatic triple-negative breast cancer compared with paclitaxel combined with placebo group^3^. The application of atezolizumab has been approved for the treatment of advanced breast cancer. Several studies have also shown that the number and co-stimulation of tumor infiltrating lymphocytes (TILs), especially CD4^+^ T, CD8^+^ T cells, and dendritic cells (DCs), are indicators of curative effects^4^. Therefore, immune regulation holds great potential in the treatment of breast cancer, and enhancement of tumor lymphocytes’ infiltration will likely show beneficial effects.

The immune system functions routinely to eliminate dead cells during normal cell turnover, infections, and injures^5^. Certain chemotherapeutic agents such as anthracyclines, oxaliplatin, and paclitaxel can trigger tumor cell death via enhanced immune destruction via eliciting the release of damage-associated molecular patterns (DAMPs) to enhance tumor immunogenicity. Immunogenic cell death (ICD) is a cell death process characterized by the upregulation of various DAMPs^6^. Calreticulin (CRT), high-mobility group protein B1 (HMGB1), adenosine triphosphate (ATP), and heat shock protein 70/90 (HSP 70/90) belong to DAMPs. As an “eat me” signal, CRT attracts antigen-presenting cells (APCs) to phagocytize the dead cells. ATP acts as a “find me” signal, which leads to immune cell infiltration into the tumor sites^7–10^.

Na^+^/K^+^-ATPase pump (NKP) is a transmembrane ion transporter expressed in various cells such as neurons and cardiomyocytes^11^. NKP serves as a multifunctional signal transducer that is essential for regulating cell apoptosis, inflammation, adhesion, and maintaining cell homeostasis^12^. NKP consists of α, β, and γ subunits. The α1 and α3 subunits of NKP are frequently overexpressed in various cancers, such as colorectal cancer, glioblastoma, and breast cancer^13,14^. Interestingly, cardiac glycosides, such as NKP inhibitors that are used for many years in the treatment of cardiac congestion, were recently showed to have potentials in the treatment of cancer^15^. Digoxin, a cardiac glycoside, has been reported to trigger ICD in osteosarcoma cells^16^. Retrospective analysis also revealed that digoxin combined with standard chemotherapy prolonged the overall survival of patients with breast cancer, colon cancer, head and neck cancer, and hepatocellular carcinoma^17^.

In the present study, we report that oleandrin, a monomer compound extracted from the leaves of *Nerium oleander* that belongs to the cardiac glycoside family^18–20^, suppresses breast cancer cell growth by inducing ICD and leading to increased immune destruction of tumor cells. The effects of oleandrin were linked to endoplasmic reticulum (ER) stress mainly through PERK/elF2α/ATF4/CHOP pathway. Our findings reveal the potential of oleandrin in cancer treatment via regulating anti-tumor immune activation.

## Materials and methods

### Reagents

Oleandrin was purchased from MedChemExpress (Monmouth Junction, NJ, USA). The protein kinase R-like ER kinase (PERK)-selective inhibitor GSK2606414 and the inositol requiring enzyme 1α (IRE1) inhibitor 4μ8C were obtained from Selleck Chemicals (Houston, TX, USA).

### Cell lines

Human breast cancer cell lines MDA-MB-231, MCF7, and T47D were obtained from the Cell Bank of the Chinese Academy of Sciences (Shanghai, China). MDA-MB-231 cells were cultured in L-15 medium. MCF7 cells were cultured in minimum Eagle’s medium. T47D cells were cultured in RPMI-1640 medium. Cell culture medium was obtained from Hyclone (GE Healthcare Life Sciences, Logan, UT, USA). Mouse breast cancer cell lines EMT6 were obtained from ATCC and cultured in Waymouth’s MB 752/1 medium (Biological Industries, Kibbutz Beit-Haemek, Israel). All the media were supplemented with 10% fetal bovine serum (FBS, Gibco, Thermo Fisher Scientific, Inc., Waltham, MA, USA). All cell lines were routinely tested for mycoplasma contamination.

### DC culture and CD8^+^ T cell isolation

Two female volunteers, aged 27 and 35, were recruited and they provided the informed consents. Peripheral blood was stained with PerCP HLA-A2 (307627, Biolegend, San Diego, CA, USA) and human leukocyte antigen (HLA) subtype was detected by flow cytometry. Peripheral blood mononuclear cells (PBMCs) were isolated from HLA-A2-type volunteer and cultured in 75 cm^2^ flask for 1 h. Suspended cells were removed, and the adherent cells were cultured with fresh X-VIVO 15 medium (Lonza, Alpharetta, GA, USA) containing 5% plasma supplemented with 500 U/ml of IL-4 and 1000 U/ml of GM-CSF (Promega, Madison, WI, USA). After 5 days of culture, DCs were collected for following experiments. CD8^+^ T cells were isolated from PBMCs using the CD8^+^ T Cell Isolation Kit according to manufacturer’s instructions (Miltenyi Biotec, CA, USA).

### Immunofluorescence staining for CRT

Breast cancer cells were treated with oleandrin. The concentrations of oleandrin used against MCF7 and MDA-MB-231 cells were 15 and 25 nM, respectively. After 24 h of treatment, cells were fixed with 95% ethanol, permeabilized with PBS containing 1% Triton X-100, and blocked using 1% BSA. Cells were incubated with anti-CRT antibody (anti-calreticulin, monoclonal rabbit; 1:75, ab2907, Abcam, Cambridge, MA, USA) at 4 °C overnight. Cells were washed 3 times with PBS and incubated with secondary antibody (goat anti-rabbit Alexa Fluor 488, 1:200, ab150077, Abcam) for 30 min. Nucleus was stained with 10 μg/ml of Hoechst 33342. Samples were finally observed under a fluorescent microscope (CQ1, Yokogawa, Japan). The cell membrane and intracellular expressions of CRT were quantified with ImageJ software (version 1.51j8, National Institutes of Health and University of Wisconsin, Bethesda, MD, USA).

### In vitro cytotoxicity assay

First, 2 × 10^4^ of MDA-MB-231 cells were seeded in a 6-well plate and divided into the following 5 groups: cells co-cultured with DCs; cells co-cultured with CD8^+^ T cells; cells pretreated with oleandrin; pretreated cells and co-cultured with DCs; and pretreated cells co-cultured with DCs and CD8^+^ T cells. MDA-MB-231 cells alone were used as control group. After 48 h of co-culture, cells were washed twice with PBS to remove the immune cells and oleandrin. Cells were continued to grow for 14 days. Finally, cells were fixed and stained with crystal violet for 15 min. The number of colonies was counted.

### Enzyme-linked immunosorbent assays (ELISA)

Breast cancer cells were treated with oleandrin. The concentrations of oleandrin used against MCF7 and MDA-MB-231 cells were 15 and 25 nM, respectively. Culture supernatant was collected and secreted ATP (Promega, Madison, WI, USA) and HMGB1 (Signalway Antibody, MD, USA) were detected with ELISA kits according to the manufacturer’s instructions. MDA-MB-231 cells pretreated with oleandrin or DMSO were co-cultured with DCs for 48 h. IL-2, IL-10, and IFN-γ from the culture supernatant were quantified using Human IL-2 Quantikine ELISA Kit (D2050), Human IL-10 Quantikine ELISA Kit (D1000B), and Human IFN-γ Quantikine ELISA Kit (DIF50C) according to the manufacturer’s instructions.

### Mouse model

Female BALB/C mice (5 week old) were obtained from Vital River Laboratories (Beijing, China). First, 2 × 10^5^ of EMT6 were inoculated into mammary fat pads of BALB/c mice. After 7 days, both long and short diameters of the tumors reached about 5 mm. The mice were randomly divided into 3 groups with 5 mice in each group, which were not blinded to investigators: PBS as control group, oleandrin treatment with 0.3 mg/kg, and 0.6 mg/kg groups. Oleandrin was administered intraperitoneally every day. Tumor volume was measured every day and quantified as 0.5 × length × width × width. After 7 days of administration, mice were sacrificed and tumors were weighed. Tumor primary cells and splenocytes were harvested for flow cytometric analysis.

### Immunohistochemistry (IHC)

Mouse tumor samples were fixed in 4% paraformaldehyde and embedded in paraffin. Tissue slices were deparaffinized, rehydrated, and antigen retrieval was performed using 10 mM citrate buffer (pH 6.0). CD80 (polyclonal rabbit, 1:200, ab215166, Abcam), CD86 (monoclonal rabbit, 1:200, ab243887, Abcam), and CD69 (polyclonal rabbit, 1:250, A00529-2, Boster) were stained overnight at 4 °C. The samples were washed and then incubated with goat anti-rabbit biotinylated secondary antibody (1:1000, ab6720, Abcam) and visualized using a horseradish peroxidase (HRP)-conjugated ABC system (1:10,000, ab7403, Abcam).

### Flow cytometric analysis

1 × 10^6^ of cells were collected and suspended in 100 μl of PBS. Cells were incubated with the following antibodies: 5 μl/test of each at room temperature for 30 min, PerCP anti-human HLA-DR, APC anti-human CD11c (301614, Biolegend), FITC anti-human CD80 (305206, Biolegend), and PE anti-human CD86 (305406, Biolegend).

Mouse primary tumor cells were minced and disassociated with the EZ enzyme (Nitta Gelatin Inc., Osaka, Japan). Single-cell suspensions from mouse spleen and tumor sites were incubated with TruStain FcX™ (anti-mouse CD16/32,101320, Biolegend) antibody to block non-specific staining and then stained on ice for 30 min with the following combination: CD3-FITC (100203, Biolegend)/CD4-PE (100407, Biolegend)/CD8-APC (100711, Biolegend), CD45-PerCP (103130, Biolegend)/CD11b-FITC (101025, Biolegend)/CD11c-APC (117309, Biolegend). Samples were detected by BD Accuri C6 (BD Biosciences, San Jose, CA, USA).

### Western blotting

Cells were collected and lysed using radio immunoprecipitation assay (RIPA) buffer. Cell culture supernatant was centrifuged for 30 min with 10,000*g* and protein concentration was measured using the protein concentration kit (2772 T, Thermo Scientific, Shanghai, China). Protein was separated by 10% sodium dodecyl sulfate polyacrylamide gel electrophoresis (SDS-PAGE) and transferred to PVDF membrane. Primary antibodies were diluted and incubated at 4 °C overnight. The membranes were then washed and incubated with secondary antibodies at room temperature for 1 h (goat anti-mouse IgG-horseradish peroxidase (HRP) (1:15,000, ab205719, Abcam) or goat anti-rabbit IgG-HRP (1:10,000, ab6721, Abcam)). Chemiluminescence was performed using Supersignal West Pico plus (Thermo Fisher Scientific, Inc.) and detected by BIO-RAD GelDoc XR + system (Bio‑Rad, Berkeley, CA, USA). Data were analyzed by Image Lab (version 5.2.1).

Primary antibodies were used as follows:

Anti-HSP70 (1:1000, 4876, Cell Signaling Technology, Danvers, MA, USA), anti-ATF3 (1:1000, 33593, Cell Signaling Technology), anti-β-actin (1:1000, 3700, Cell Signaling Technology), anti-PERK (1:500, ab79483, Abcam), anti-eIF2α (1:500, ab5369, Abcam), anti-elF2α (phosphor S52, 1:1000, ab227593, Abcam), anti-ATF4 (1:1000, ab23760, Abcam), anti-CHOP (1:200, ab11419, Abcam), anti-PERK (phospho T982, 1:1000, ab192591, Abcam), anti-IRE1 (1:1000, ab37073, Abcam), anti-IRE1 (phosphor S724, 1:1500, ab124945, Abcam), anti-XBP1 (1:1000, ab37152, Abcam), anti-GADD34 (1:2000, ab9869, Abcam), and anti-HSP90 antibody (1:200, sc-69703, Santa Cruz Biotechnology, Dallas, TX, USA).

### RNA sequencing

MCF7, MDA-MB-231, and T47D cells were treated with oleandrin for 10 h. The concentrations of oleandrin used against MCF7, MDA-MB-231, and T47D cells were 15, 25, and 12.5 nM, respectively. Total RNA of treated cells and controls were extracted using TRIzol. The mRNA was sequenced using the Illumina Hiseq platform. Differential expression analysis of 2 groups was performed using the DESeq2 R package (1.16.1). The data were transformed into Venn’s diagrams and heatmap. Gene Ontology (GO) and KEGG enrichment analysis of differentially expressed genes was implemented by the cluster Profiler R package.

### Quantitative real-time polymerase chain reaction (qRT-PCR)

Cells were lysed with 1 ml of TRIzol reagent (Thermo Fisher Scientific, Carlsbad, CA, USA), and cDNA was synthesized with the PrimeScript™RT reagent Kit (TaKaRa, Shiga, Japan). qRT-PCR was performed by using SYBR Premix EXtaq (TaKaRa, Shiga, Japan). Primers used are as follows:

CD86: forward, 5′-CTGCTCATCTATACACGGTTACC-3′

reverse, 5′-GGAAACGTCGTACAGTTCTGTG-3′

CD80: forward, 5′-GGCCCGAGTACAAGAACCG-3′

reverse, 5′-TCGTATGTGCCCTCGTCAGAT-3′

IL-2: forward, 5′-TCCTGTCTTGCATTGCACTAAG-3′

reverse, 5′-CATCCTGGTGAGTTTGGGATTC-3′

IL-10: forward, 5′-TCAAGGCGCATGTGAACTCC-3′

reverse, 5′-GATGTCAAACTCACTCATGGCT-3′

IFN-γ: forward, 5′-TCGGTAACTGACTTGAATGTCCA-3′

reverse, 5′-TCGCTTCCCTGTTTTAGCTGC-3′

GAPDH: forward, 5′-ACAACTTTGGTATCGTGGAAGG-3′

reverse, 5′-GCCATCACGCCACAGTTTC-3′

### Statistical analysis

Statistical analysis was performed using the Statistical Package for the Social Sciences (SPSS) software version 21 (IBM Corp., Armonk, NY, USA). Experiments were routinely performed with three biological repeats unless specified. The results were represented as mean ± standard deviation of the mean from three independent experiments. The comparison between the 2 groups was conducted by *t*-test or one-way ANOVA. *p* < 0.05 was considered statistically significant (*p < 0.05, **p < 0.01).

## Results

### Oleandrin induces ICD in breast cancer cells in vitro

**Fig. 1:**
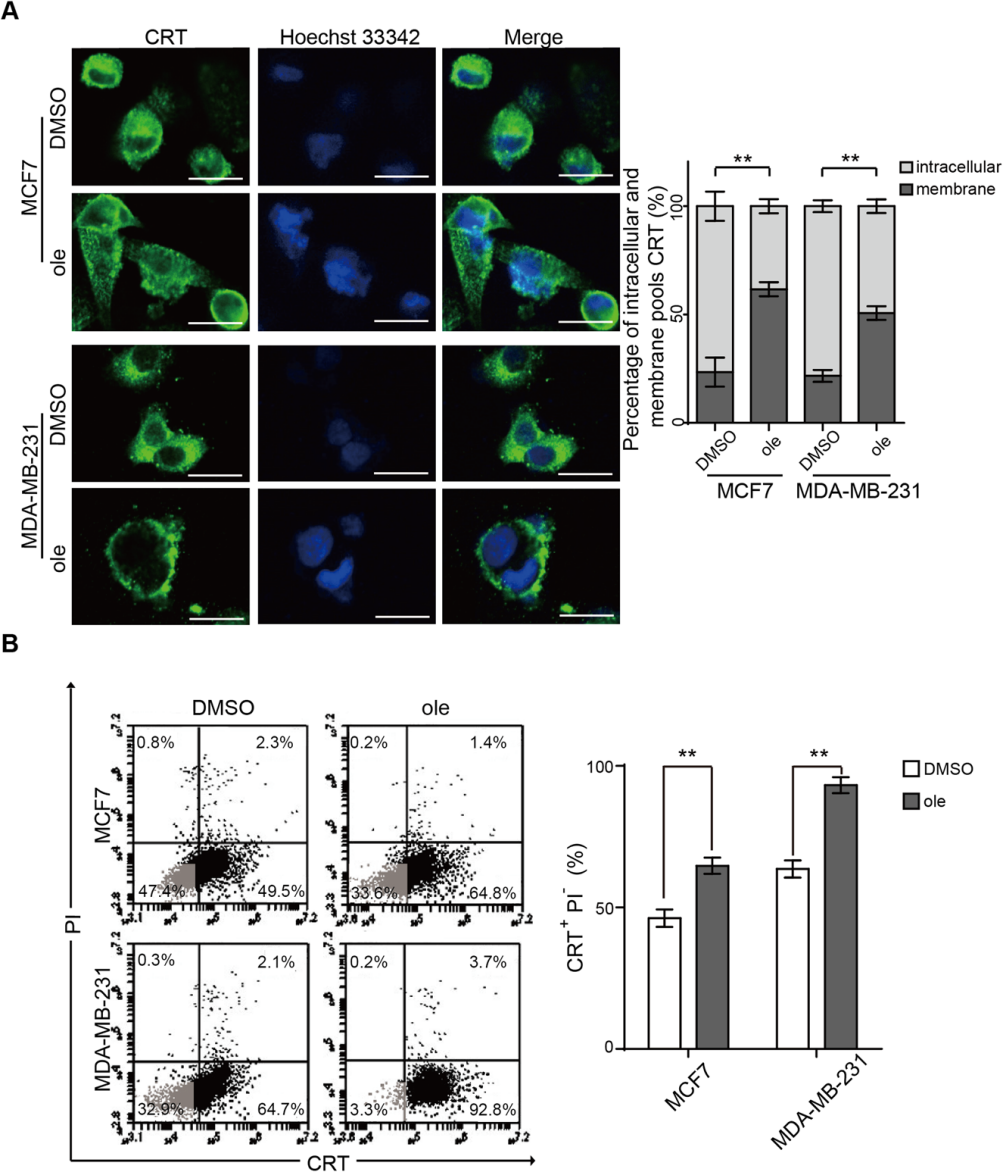
Oleandrin induces CRT exposure in breast cancer cells in vitro. MCF7 and MDA-MB-231 cells were treated with oleandrin for 10 h. **A** Immunofluorescence analysis of cells stained with CRT and Hoechst 33342. Scale bar = 20 μm. Intensities of the cell membrane and intracellular expressions of CRT were quantified with ImageJ software (Version 1.51j8) and plotted. **B** Cells were stained with CRT and PI, and detected by flow cytometry. The CRT-positive and PI-negative cells were showed in representative dot plots and quantification data. Data are presented as mean ± standard error of the mean from three independent experiments (*p < 0.05). ole, oleandrin.

**Fig. 2:**
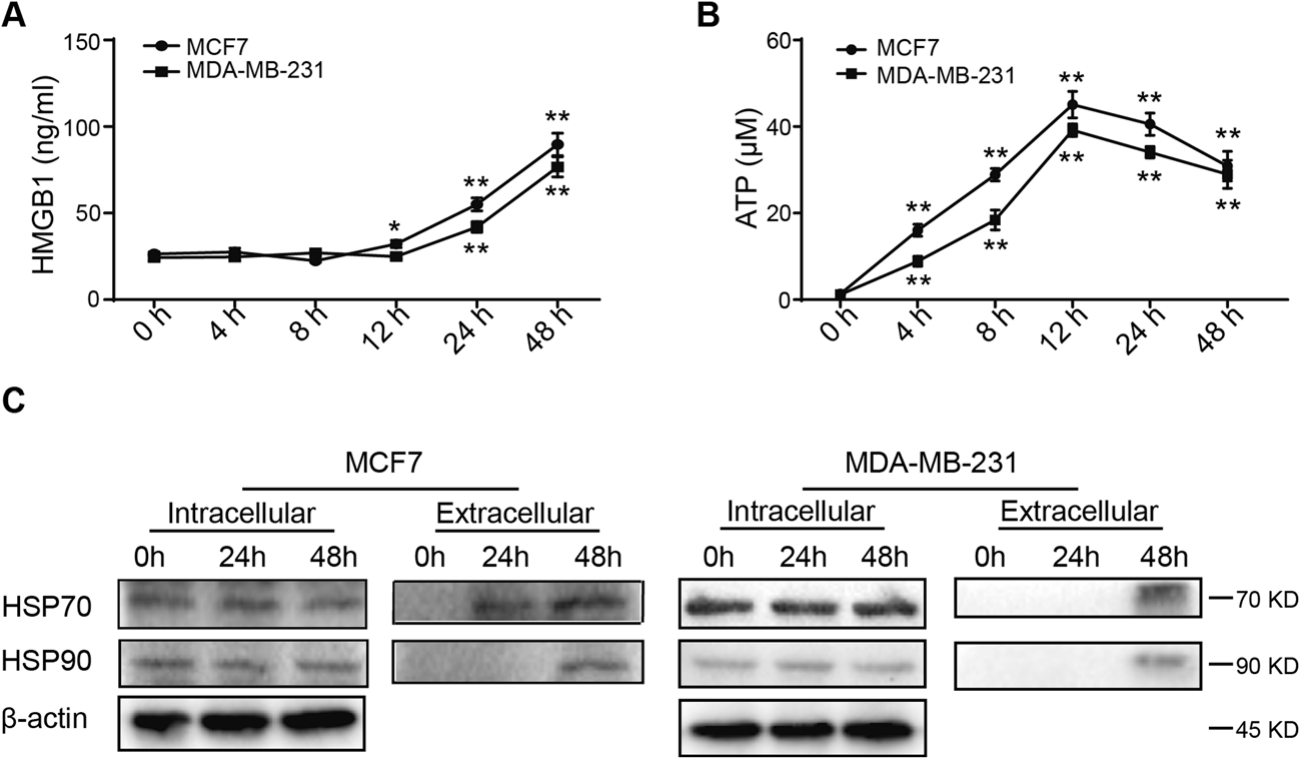
Oleandrin induces HMGB1 and ATP releasements. MCF7 and MDA-MB-231 cells were treated with oleandrin and culture supernatants were collected 0, 4, 8, 12, 24, and 48 h after treatment. **A** Secreted HMGB1 was detected by ELISA. **B** ATP secretion was detected by chemiluminescence assay. Data were acquired from three independent experiments. *p < 0.05, **p < 0.01 vs. 0 h cells. **C** Cell supernatants were collected and concentrated at 0, 24, and 48 h after oleandrin treatment. At the same time cell lysates were collected. The expression of HSP70 and HSP90 were detected by western blotting.

Our previous study has established the IC50 concentrations of oleandrin against MCF7 and MDA-MB-231 cells, which were 14.5 and 24.62 nM, respectively^21^. Therefore, the treatment concentrations of MCF7 and MDA-MB-231 cells were used at 15 and 25 nM in the following experiments. CRT exposure on cell surface, a marker for ICD, is an “eat me” signal to stimulate DC maturation and immune activation. To investigate whether oleandrin induces ICD in breast cancer, MCF7, and MDA-MB-231 cells were treated with oleandrin for 10 h before immunofluorescence assays to stain CRT. As shown in Fig. 1A, compared to the control, oleandrin treatment led to increased CRT expression on the cell membrane. Cell surface expression of CRT was further confirmed by flow cytometry analyses. The proportion of PI^−^/CRT^+^ subpopulation was compared between the control and oleandrin-treatment groups (Fig. 1B). Consistent with immunofluorescence results, oleandrin treatment increased cell surface CRT expression in both MCF7 and MDA-MB-231 cells. The release of other DAMPs, including HMGB1, ATP, and HSP70/90, was also compared between the control and oleandrin-treatment groups. The secreted HMGB1 was detected after 12 h and increased significantly at 24 and 48 h in MCF7 cells following oleandrin treatment. No significant release of HMGB1 was observed in MDA-MB-231 cells at 12 h, but increased significantly at 24 and 48 h (Fig. 2A). Distinct from the results of HMGB1 release, ATP secretion was observed 4 h after oleandrin treatment in both MCF7 and MDA-MB-231 cells and reached the peak at 12 h (Fig. 2B). Moreover, both the intracellular and extracellular expressions of HSP70/90 were detected, as presented in Fig. 2C, intracellular expressions of HSP70/90 were not affected by oleandrin in MCF7 and MDA-MB-231 cells. However, the extracellular expressions of HSP70 and HSP90 were detected in both cells at 48 h after oleandrin treatment. These data indicated that oleandrin treatment triggered ICD in breast cancer cells.

### Oleandrin induces ICD-associated immune activation in vitro

**Fig. 3:**
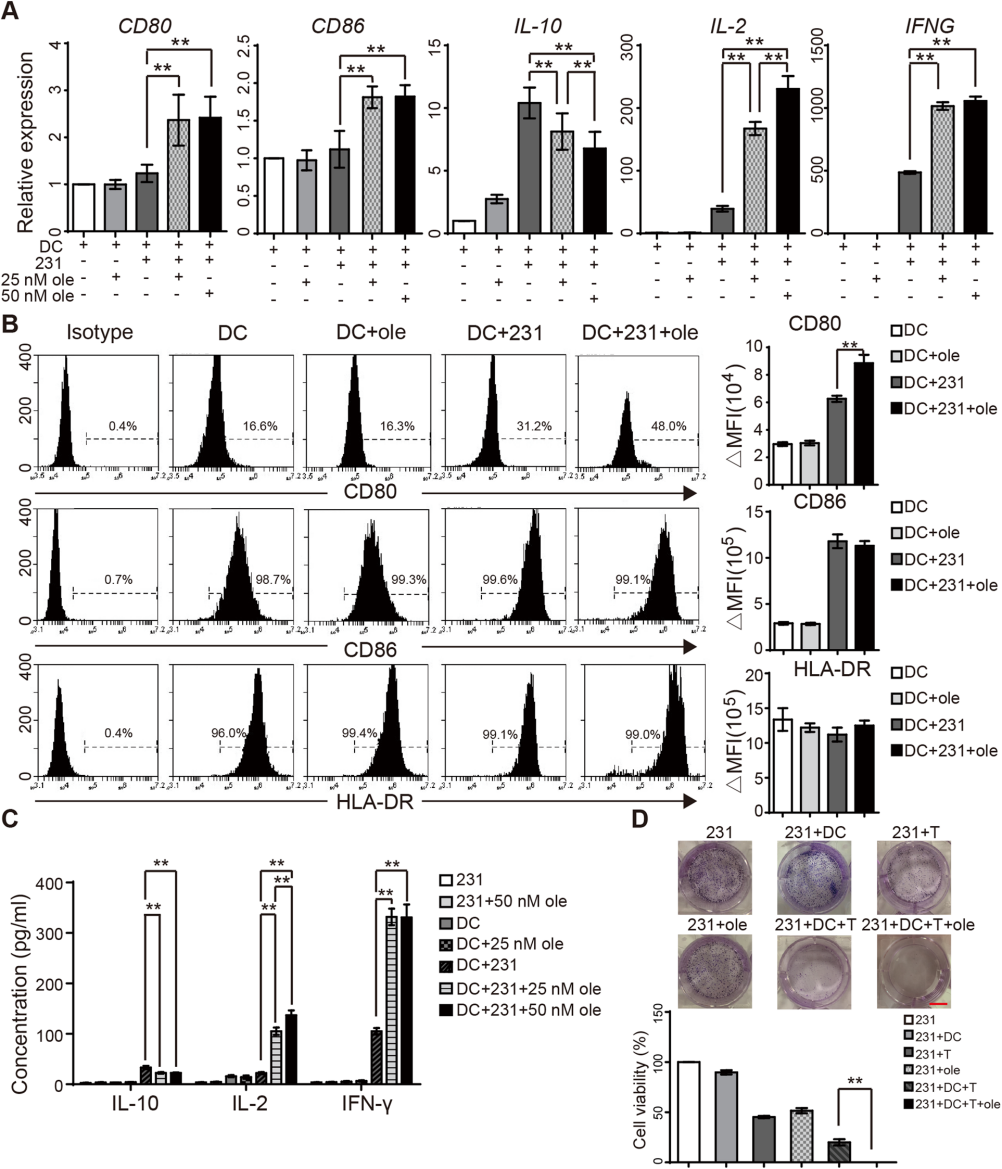
Oleandrin induces ICD-associated immune activation in vitro. DCs were co-cultured with MDA-MB-231 with or without oleandrin treatment for 48 h. DCs were sorted by staining with CD45. **A** The relative expression of *CD80*, *CD86*, *IL-10*, *IL-2*, and *IFNG* in DCs were detected by qRT-PCR. **B** The expression of CD80, CD86 and HLA-DR on DCs surface were further detected by flow cytometry. Cells stained with isotype antibody were used as control. Data were represented by ΔMFI values. **C** MDA-MB-231 cells were pretreated with oleandrin for 24 h and co-cultured with DCs for further 48 h. Cytokine secretion in the culture supernatant was detected by ELISA. **D** The cell growth of the following groups was determined by clone-formation assay: MDA-MB-231, MDA-MB-231/DCs, MDA-MB-231/CD8^+^ T cells, MDA-MB-231/DCs/CD8^+^ T cells, 25 nM oleandrin-treated MDA-MB-231, and 25 nM oleandrin-treated MDA-MB-231/DCs/CD8^+^ T cells. The representative pictures (upper) and quantification data (lower) were shown. Data are presented as mean ± standard error of the mean from three independent experiments. **p < 0.01. 231, MDA-MB-231; ole, oleandrin.

Tumor cell ICD is capable of inducing antigen-presenting function of DCs. As a result, a series of immune responses are activated, including cytokine secretion and T cell activation^22^. To eliminate the uncertainty caused by different HLA, we detected the HLA subtypes of MCF7 and MDA-MB-231 cells in two volunteers. As shown in sFig. 1A, B, HLA subtype of MDA-MB-231 was HLA-A2 that matched that of the volunteers. To investigate whether oleandrin treatment induces DC maturation and activation, we cultured DCs isolated from PBMC and then co-cultured with MDA-MB-231 cells with or without oleandrin pretreatment. The morphologic features of DCs were observed, which showed that the adherent cells were round or oval on day 1 and extended branched projections on day 5 (sFig. 1C). Flow cytometry data confirmed that 99.37 ± 0.15% of cells were CD11c-positive, which indicated mature DCs on day 5 (sFig. 1D). DCs were co-cultured with MDA-MB-231 cells at a ratio of 1:1 with or without oleandrin pretreatment. After 48 h of co-culture, DCs were stained with CD45 and separated by a cell sorter (sFig. 1E). DC maturation and activation markers (CD80, CD86, and cytokine expression in DCs) were detected by qRT-PCR. The expression levels of CD80, CD86, IL-10, IL-2, and IFN-γ genes were not affected by oleandrin alone. However, co-culture of DCs with MDA-MB-231 cells increased the expression of IL-10, IL-2, and IFN-γ, but had no effects on CD80 and CD86 expressions. Compared with DC/MDA-MB-231 group, DCs co-cultured with oleandrin-pretreated MDA-MB-231 cells showed significantly enhanced levels of CD80, CD86, IL-2, and IFN – γ but decreased IL-10 expression. The effects on IL-2 and IL-10 were dose-dependent on oleandrin (Fig. 3A, **p < 0.01). The expressions of PD-L1 and Tim-3 on DCs were not changed by oleandrin treatment (data not shown).

Cell surface expressions of CD80, CD86, and HLA-DR were detected by flow cytometry and represented by Δ median fluorescence intensity (ΔMFI). Cells stained with isotype control were considered as control (Fig. 3B). IL-2, IL-10, and IFN-γ expressions in the co-culture supernatant were further confirmed by ELISA (Fig. 3C). Consistent with the qRT-PCR results, DCs co-cultured with oleandrin-pretreated MDA-MB-231 cells showed significantly enhanced levels of CD86, IL-2, and IFN-γ but decreased IL-10 expression. Taken together, these results suggest that co-culture with oleandrin-treated breast cancer cells significantly enhanced DC maturation and activation.

DCs, as APCs, cross-present tumor-associated antigens to cytotoxic CD8^+^ T lymphocytes^23^. To investigate whether oleandrin treatment enhances DC-mediated anti-tumor response, MDA-MB-231 cells treated with oleandrin were co-cultured with DCs and CD8^+^ T cells, and cell growth were detected by colony-formation assay. Cell experiment groups were divided as follows: MDA-MB-231/DCs, MDA-MB-231/CD8^+^ T cells, pretreated MDA-MB-231, MDA-MB-231/DCs/CD8^+^ T cells, oleandrin-pretreated MDA-MB-231/DCs/CD8^+^ T cell, and cell growth was 89.8 ± 2.14%, 45.24 ± 1.19%, 51.11 ± 2.60%, 19.97 ± 3.12%, 0.01 ± 0.0017%, respectively (Fig. 3D). Therefore, pretreatment with oleandrin enhanced DC-mediated T cell cytotoxicity in vitro.

### Oleandrin suppresses tumor growth in mouse model

**Fig. 4:**
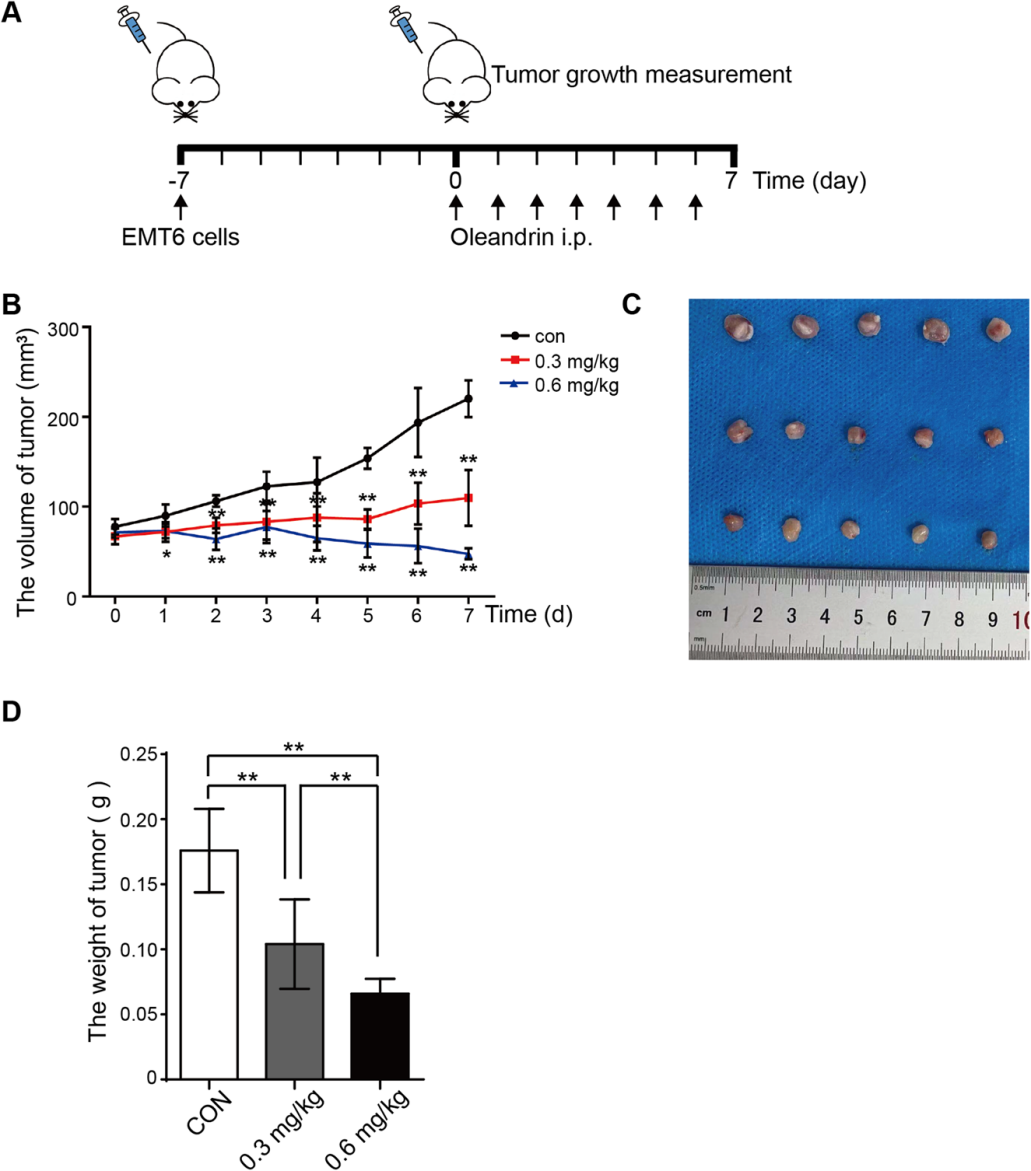
Oleandrin suppresses tumor growth in mice. **A** Diagram of procedure for mouse tumor model. First, 2 × 10^5^ EMT6 cells were transplanted into mammary fat pads of BALB/c mice. Seven days after transplantation, tumor-bearing mice were divided into 3 groups and treated with oleandrin at 0.3 and 0.6 mg/kg intraperitoneal for 7 days. Mice treated with PBS were used as control. **B** Tumor volume was measured every day and quantified as 0.5 × length × width × width. The volume was expressed as mean ± SD (*n* = 5) and represented as tumor volume–time curves to show (*p < 0.05, **p < 0.01 vs. control.). **C** After 7 days of administration, mice were sacrificed, and tumors were weighted. The representative tumors were shown. **D** Tumor weight in each group was expressed as mean ± SD (*n* = 5, **p < 0.01 vs. control).

To investigate the anti-tumor effects of oleandrin in vivo, BALB/c mice were used to implant EMT6 cells into mammary fat pads. Seven days after implantation, tumor-bearing mice were divided into 3 groups and treated with oleandrin at 0.3 and 0.6 mg/kg intraperitoneal. Mice treated with vehicle were used as control (Fig. 4A). Compared with the control group, tumor growth was inhibited in the oleandrin-treatment groups 1 day after administration. After 7 days of continuous administration, the average tumor size of 0.3 mg/kg treatment group was unchanged compared with day 0, while the average tumor size of 0.6 mg/kg treatment group was even smaller than that at day 0 (Fig. 4B, *p < 0.05, **p < 0.01). The average tumor weight of 0.6 mg/kg treatment group was 1.58 times lower than that of 0.3 mg/kg treatment group and was 2.66 times lower than that of the control group (Fig. 4C, D, **p < 0.01).

### Oleandrin activates anti-tumor immune responses in mice

**Fig. 5:**
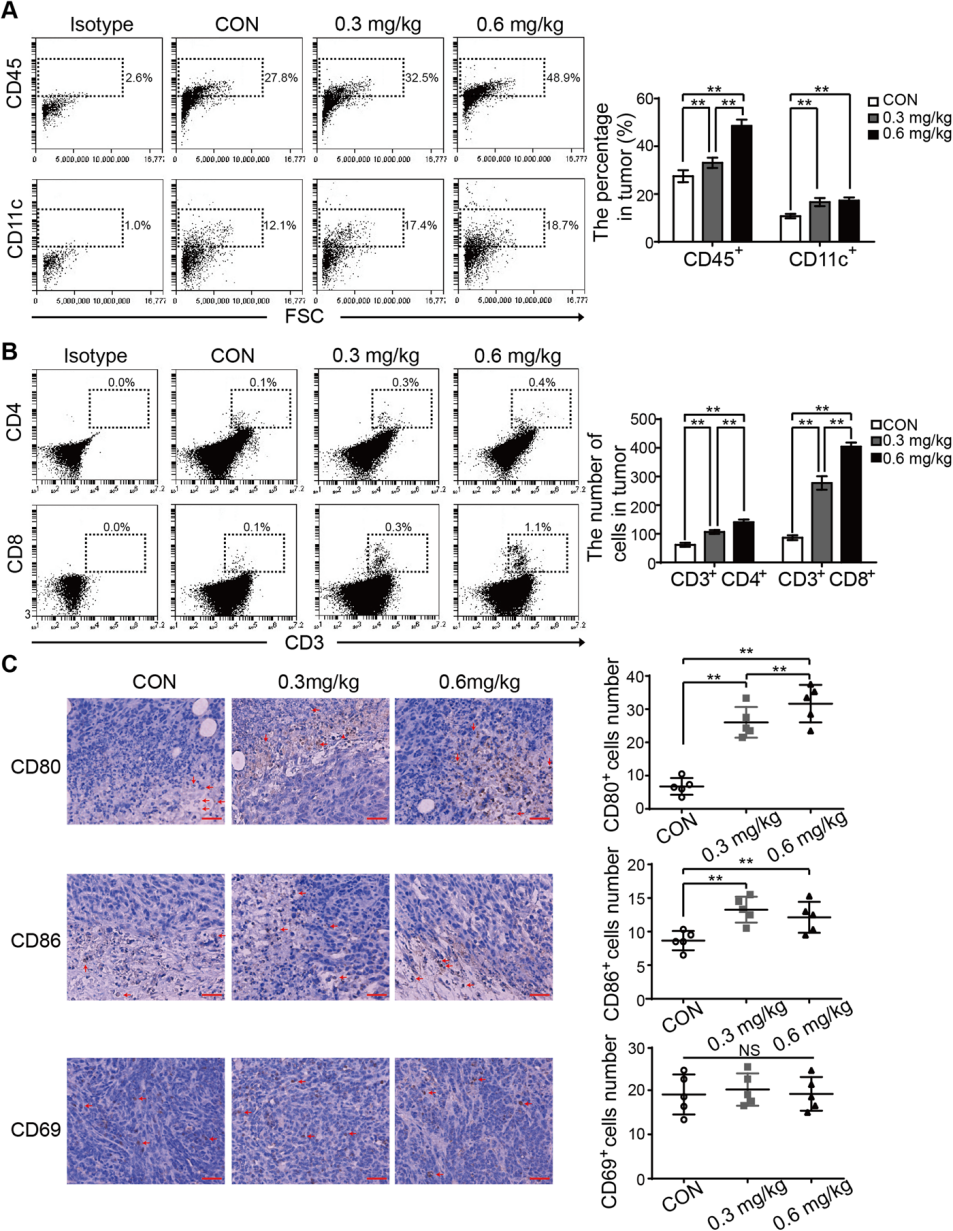
Oleandrin activates anti-tumor immune response in mice. Tumor primary cells were collected from the tumor-bearing mice and detected by flow cytometry. **A** TIL and tumor infiltrating DCs were analyzed by staining with CD45 and CD11c. The results were represented as percentage in tumors. **B** Tumor infiltrating T cells were analyzed by staining with CD3, CD4, and CD8. The absolute numbers of CD4^+^ and CD8^+^ T cells in tumors were expressed as mean ± SD. **C** Mouse tumor samples were stained using IHC for CD80, CD86, and CD69 expressions. Cell numbers were counted under a 40× objective. Each dot denotes the immune cell number from a tumor section. Mean and standard deviation of immune cell number in each group are shown. Scale bar = 40 μm. *n* = 5, **p < 0.01 vs. control. CON, control.

To investigate the alterations in the immune microenvironment after oleandrin treatment, tumor primary cells and splenocytes were collected and stained with T cell and DC markers. The TILs were analyzed by staining with CD45. Compared with the control group, oleandrin treatment increased the portion of CD45^+^ cells in a dose-dependent manner. Moreover, DC (CD45^+^/CD11c^+^) were also increased by oleandrin treatment (Fig. 5A, **p < 0.01). Compared with CD45^+^ cells and CD11c^+^ cells, the proportion of tumor infiltrating T cells was much less. Therefore, the absolute numbers of CD4^+^ and CD8^+^ T cells were used to compare. As shown in Fig. 5B, oleandrin treatment increased the numbers of both CD4^+^ and CD8^+^ T cells (**p < 0.01). Immune cell function plays a vital role during tumor progression in the microenvironment. CD69 was reported to act as a costimulatory molecule for T cell activation and proliferation^23^. Therefore, CD80, CD86 along with CD69 were further stained by IHC (Fig. 5C). Compared to the control group, oleandrin treatment led to increased numbers of both CD80^+^ and CD86^+^ cells, while the expression of CD69 was not affected. These data indicated that oleandrin treatment promoted DC activation and increased T and DC infiltration into the tumor sites.

Moreover, as presented in sFig. 2A, B, compared with the control group, oleandrin-treatment groups showed increased populations of both CD4^+^ and CD8^+^ T cell in spleen in a dose-dependent manner, while CD11b^+^/CD11c^+^ or CD11b^+^/CD11c^−^ cells were not changed.

### Oleandrin-induced CRT exposure is independent of caspase activity

**Fig. 6:**
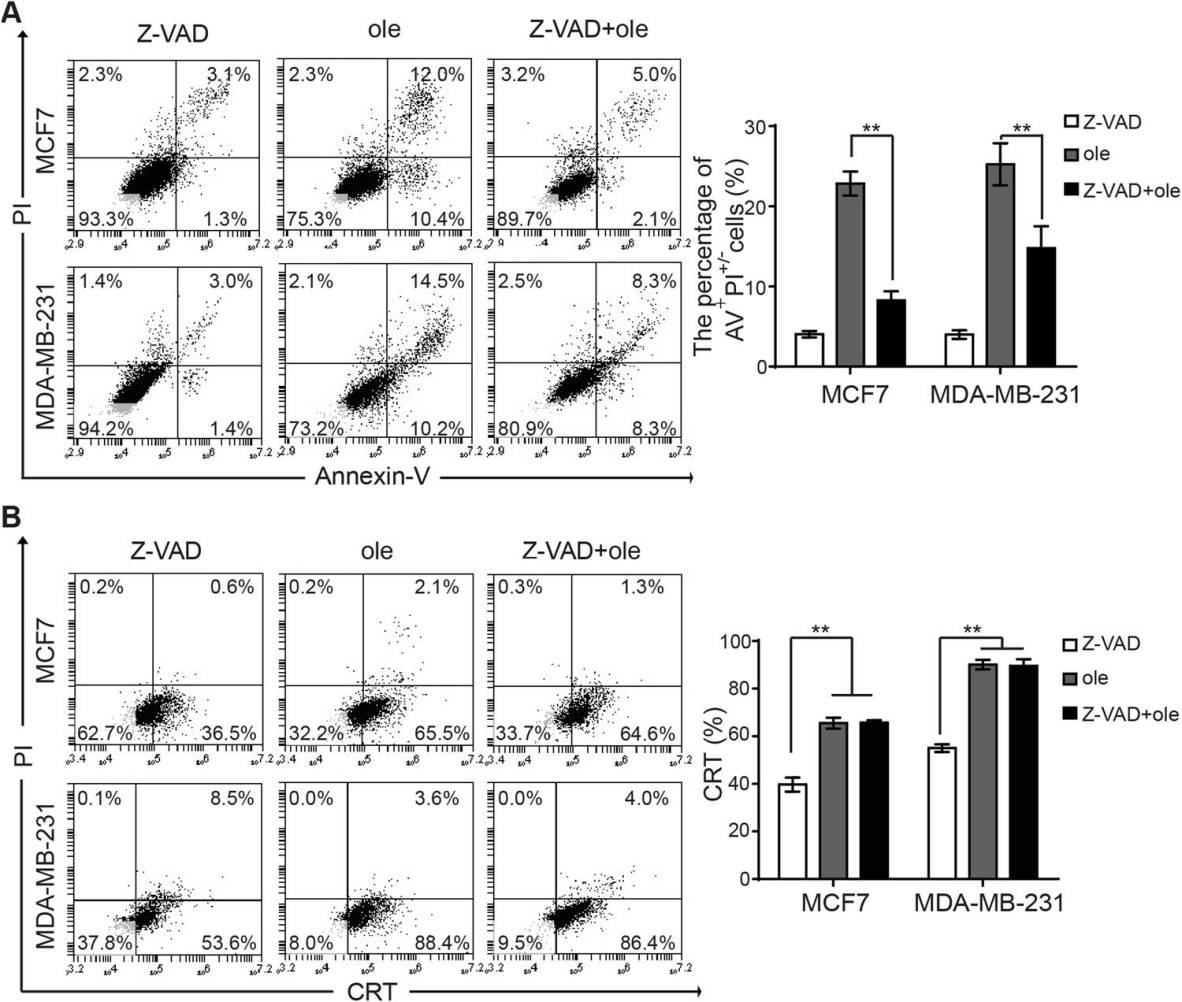
Oleandrin induces CRT exposure was independent of caspase activity. The MCF7 and MDA-MB-231 cells were pretreated with pan-caspase inhibitor Z-VAD-FMK for 10 h, then treated with oleandrin for further 24 h. **A** Cell apoptosis was detected by staining with Annexin-V and PI. The apoptosis rate (Annexin-V^+^ and PI^+/−^) was shown as mean ± SD (*n* = 3, **p < 0.01 vs. control). **B** The CRT exposure on cell surface was detected by flow cytometry. The percentage of CRT^+^ and PI^-^ cells were shown as mean ± SD (*n* = 3, **p < 0.01 vs. control). AV, Annexin-V; ole, oleandrin.

Translocation of CRT from ER to cell surface is an indicator for ICD. Previous studies have revealed that chemotherapeutic agents such as anthracyclines and oxaliplatin can induce ICD in tumor cells via caspase-8-dependent pathway. Caspase-8 hydrolyzes the ER anchor protein B cell receptor-associated protein 31 (BAP31), which further transfers CRT to cell surface through Golgi apparatus^24,25^. This procedure is essential, as blocking caspase 8 inhibits CRT exposure. Our previous study has shown that oleandrin induced breast cancer cell apoptosis. To explore whether oleandrin-induced ICD is dependent on caspase, MCF7 and MDA-MB-231 cells were pretreated with Z-VAD-FMK, a pan-caspase inhibitor, for 10 h before treatment with oleandrin. Cell apoptosis and CRT exposure were detected by flow cytometry. As shown in Fig. 6A, Z-VAD-FMK significantly inhibited breast cancer cell apoptosis. However, CRT exposure was not affected (Fig. 6B). These data indicated that oleandrin-induced CRT exposure was likely not associated with caspase.

### Oleandrin induces ICD via ER stress

**Fig. 7:**
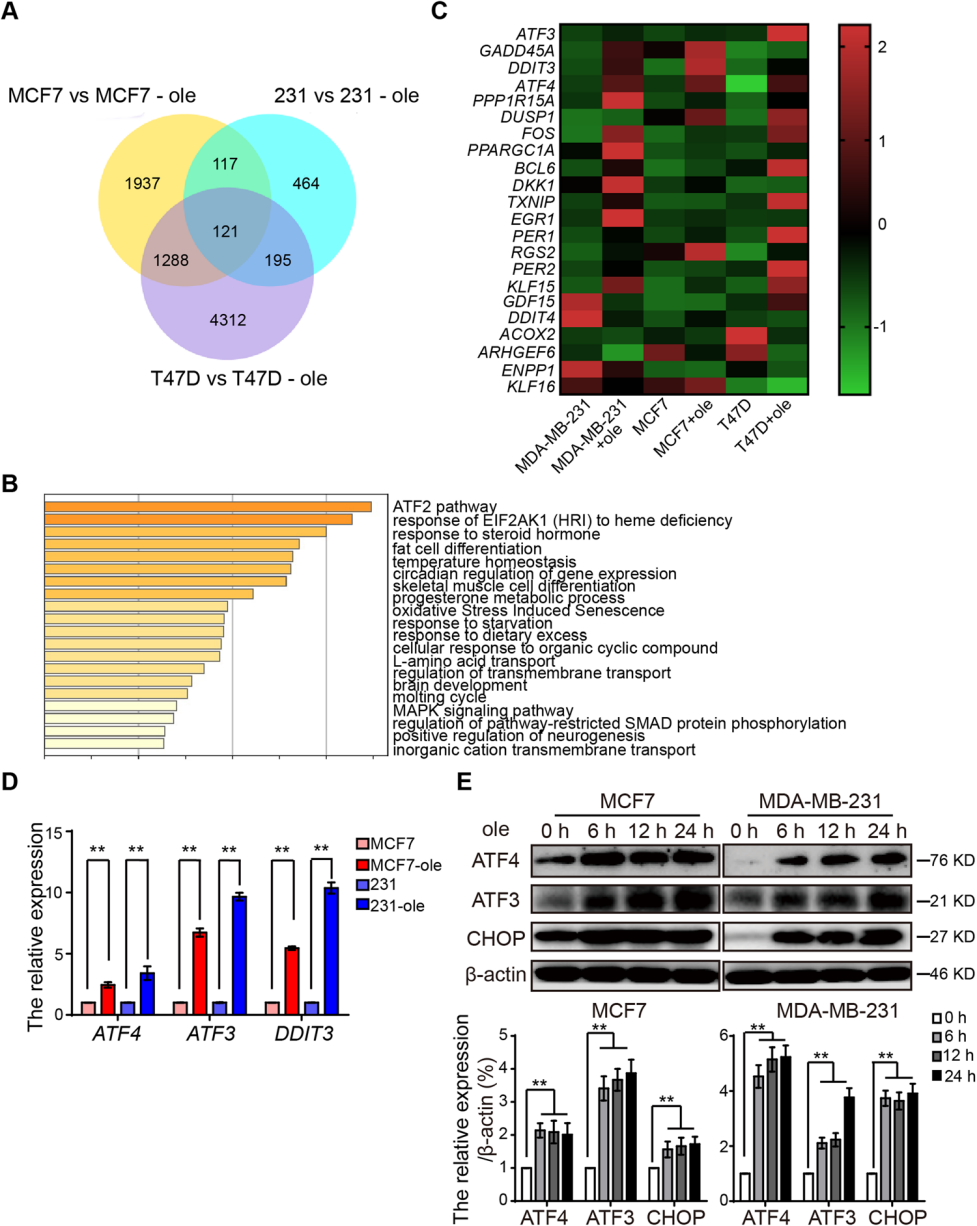
Oleandrin induces ICD via ER stress. MCF7, T47D, and MDA-MB-231 cells were treated with oleandrin or DMSO for 10 h. The differential of mRNA was analyzed by mRNA sequencing. **A** The Venn’s diagrams show the significant difference mRNA in three different pairs of cells. **B** The pathway analysis was performed functional enrichment of these different mRNA in GO and KEGG databases. **C** The heatmap was generated based on the significant different genes in ATF2 pathway. **D**, **E** The ATF3, ATF4, and CHOP (*DDIT3*) expression was detected by qRT-PCR and western blotting. Data are presented as mean ± standard error of the mean from three independent experiments. **p < 0.01. ole, oleandrin.

To further explore the mechanism of oleandrin-induced ICD in breast cancer cells, MCF7, T47D, and MDA-MB-231 cells were treated with oleandrin and differential mRNA expressions were analyzed by RNA sequencing. A total of 121 common significantly different genes in the three different pairs of cells were identified in the Venn’s diagrams (Fig. 7A). The most significantly changed genes were categorized into the ATF2 pathway (Fig. 7B). The identified genes involved in ATF2 pathway were illustrated using a heatmap. As shown in Fig. 7C, activating transcription factor 3 (*ATF3*), DNA damage inducible transcript 3 (*DDIT3*), and activating transcription factor 4 (*ATF4*) were reported as the downstream genes of the ER stress pathway. Their altered expressions were further validated by qRT-PCR (Fig. 7D). The mRNA expression levels of these three genes were confirmed to be upregulated by oleandrin treatment. Consistent with qRT-PCR results, the protein expression levels of ATF3, ATF4, and CHOP (*DDIT3*) were also increased accordingly (Fig. 7D). These data indicated that oleandrin-induced ICD might be associated with ER stress.

### Oleandrin induces ER stress in breast cancer

**Fig. 8:**
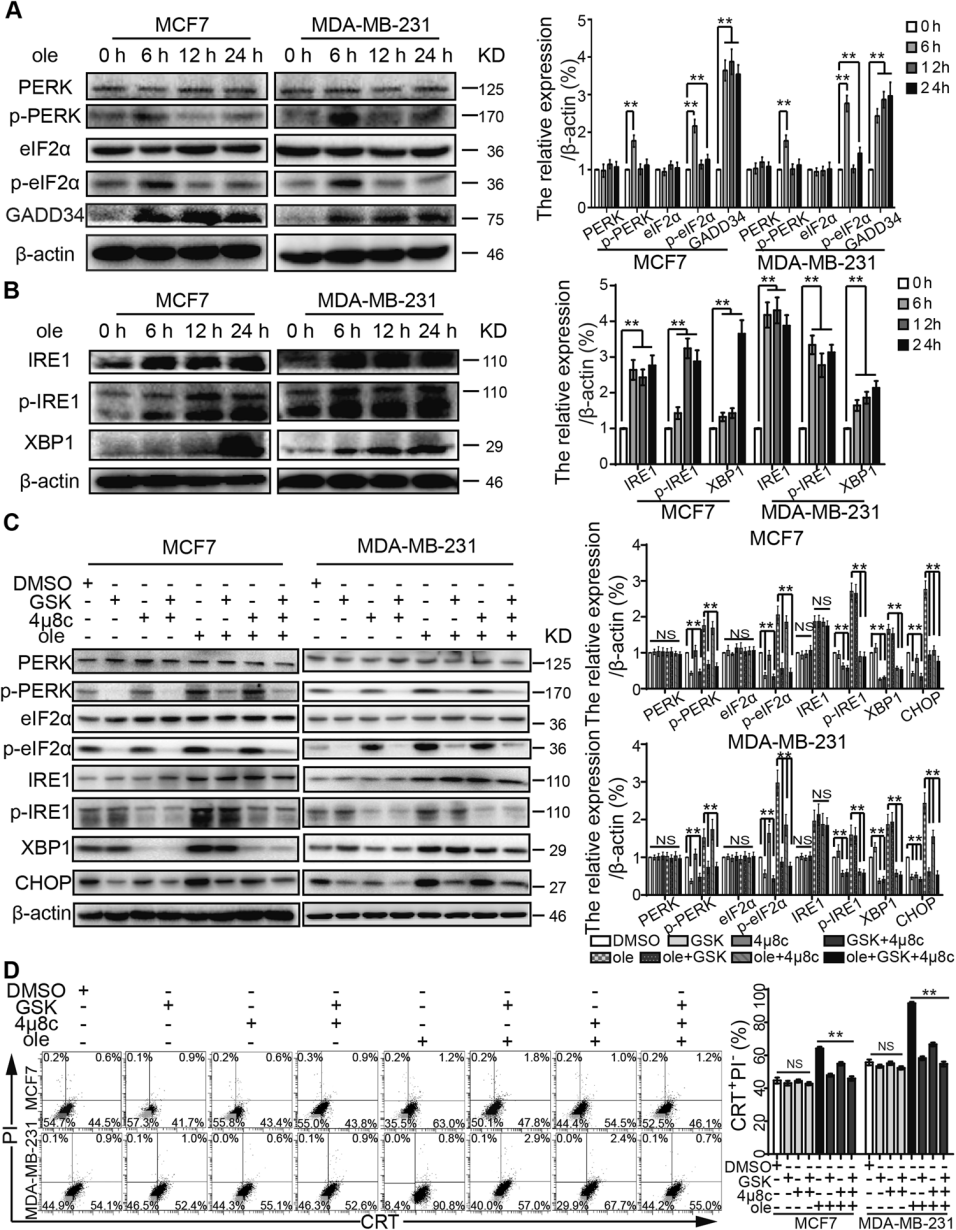
Oleandrin induces breast cancer ER stress. **A**, **B** MCF7 and MDA-MB-231 cells were treated with oleandrin for 0, 6, 12, and 24 h. The protein expressions were analyzed by western blotting. **C** MCF7 and MDA-MB-231 cells were pretreated with the PERK inhibitor GSK2606414 at 6 μM and the IRE1 inhibitor 4μ8C at 5 μM for 12 h before treatment with oleandrin for further 6 h. β-actin was used as loading control. **D** Treated cells were stained with CRT and PI before detected by flow cytometry. The CRT-positive and PI-negative cells were showed in representative dot plots and quantification data. **p < 0.01. ole, oleandrin; GSK, GSK2606414.

In mammalian cells, various cytotoxic stimuli can cause unfolded protein responses (UPRs), which function as an adaptive cellular program to sustain protein homeostasis to protect cells^26^. Such imbalance incurred by various stimuli can cause ER stress. ER stress is initiated by three ER transmembrane sensors: PERK, IRE1, and activating transcription factor 6 (ATF6). These three sensors can further activate downstream signaling pathways. The expression of total PERK, its substrate eukaryotic translation initiation factor 2α (eIF2α) and ATF6 were not affected by oleandrin treatment (Fig. 8A and sFig. 1F). However, phosphorylation levels of PERK and eIF2α (S52) were enhanced 6 h after oleandrin treatment. In addition, the activation of ATF4, and the expressions of ATF4-dependent target protein (growth-arrest- and DNA-damage-induced transcript 34 (GADD34, PPP1R15A), and CHOP) were increased as well (Fig. 8A). Previous studies have demonstrated that GADD34 associates with the broadly acting serine/threonine protein phosphatase 1 (PP1) to dephosphorylate eIF2α^27^. This may be a feedback mechanism for cell homeostasis. Moreover, the expression of IRE1 and phosphorylation of IRE1 (S724) were significantly increased, and subsequently enhanced the downstream XBP1 expression (Fig. 8B). In order to test if PERK and IRE1 acted as important mediators of oleandrin-induced ICD, MCF7 and MDA-MB-231 cells were treated with the PERK-selective inhibitor GSK2606414. As shown in Fig. 8C, D, GSK2606414+ oleandrin treatment suppressed the expressions of p-PERK, p-EIF2α, and CHOP, as well as CRT exposure. The IRE1 inhibitor 4μ8C decreased p-IRE1 and XBP1 levels but only weakly inhibited the CHOP expression and CRT exposure. The combination of GSK2606414 and 4μ8C showed the strongest inhibitory effects on CHOP expression and CRT exposure. Moreover, similar results were obtained by genetic inhibition using siRNAs targeting PERK and IRE1 (sFig. 2C, D). Taken together, oleandrin induced ICD mainly through the PERK/elF2α/ATF4/CHOP pathway. The pharmacological and genetic inhibition of PERK suppressed oleandrin-triggered ICD.

Oleandrin, as a Na^+^/K^+^ ATPase inhibitor, increases the concentration of Na^+^ and activated Na^+^/Ca^2+^ ion exchange channel on cell surface, which causes the influx of Ca^2+^. Loss of cellular homeostasis and disruption of Ca^2+^ leads to activation of ER stress-associated pathways including PERK/elF2α/ATF4 and IRE1-XBP1. ER stress enhances the releasements of ATP and HMGB1. Moreover, ER stress induces CRT exposure to the cell surface. The release of these DAMP signals eventually leads to the enhancement of immune response (sFig. 3).

## Discussion

*Nerium oleander* is widely distributed in subtropical Asia, Mediterranean coast, and southwest America. The flowers and leaves are commonly used in folk medicine for the treatment of heart failure, leprosy, malaria, ringworms, indigestion, and as analgesic and anti-inflammatory^28,29^. Oleandrin is a monomer compound extracted from the leaves of *Nerium oleander* that contains potent cardenolides, which shows strong heart-related clinical effect^30,31^. Recent studies have revealed that oleandrin has anti-tumor activities in various tumors such as ovarian cancer, glioma, colon cancer, osteosarcoma, bladder cancer, breast cancer, and leukemia. The working mechanism involves the suppression of Akt phosphorylation and the inhibition of mTOR^32–36^. The botanical drug candidates of oleandrin, Anvirzel™ and PBI-05204, have been tested in phase I clinical trials for the treatment of solid tumors. Pbi-05204 was administered under a dose of 0.2255 mg/kg/day, and no side effects above grade 3 occurred, which proved its safety in patients with advanced cancer^37^. Our previous study demonstrated that oleandrin had obvious cytotoxic effects on MCF7 (Luminal A subtype), SK-BR-3 (HER2^+^ subtype), and MDA-MB-231 (TNBC subtype) cells, but no obvious inhibitory effect on MCF10A was observed^21^.

DCs are potent APCs and play an important role in ICD-mediated immune response. In this study, we show that DC maturation and activation were not affected by oleandrin treatment. However, CD80 expression increased significantly on DCs co-cultured with oleandrin-pretreated MDA-MB-231 cells. CD80 along with CD86 that are markers of active DCs bind to CD28 on T cells and act as a costimulatory signal for T cell activation^38^. Moreover, oleandrin treatment decreased IL-10 expression but increased IL-2 and IFN-γ secretion. As an immunosuppressive cytokine, IL-10 inhibits the function of DC cells and weakens the immune activities of CD4^+^ and CD8^+^ T cells^39^. IL-2 plays an essential role for the proliferation of antigen-specific T cells^40^. IFN-γ induces the differentiation of Th1 and NKT cells and is important for anti-tumor immunity^41^. These data indicate that oleandrin triggered ICD and activated DC-mediated immune responses. Consistent with our hypothesis, cytotoxic activities of CD8^+^ T cells were enhanced by oleandrin pretreatment in vitro. In vivo experiments further confirmed that intraperitoneal administration of oleandrin at both 0.3 and 0.6 mg/kg inhibited tumor growth. Oleandrin treatment increased the number of tumor infiltrating CD45^+^ cells including CD11c^+^ DCs, CD4^+^ T cells, and CD8^+^ T cells. The infiltrations of CD4^+^ and CD8^+^ T cells were dose-dependent, especially in CD8^+^ T cells. Furthermore, we analyzed T cells in tumor-bearing mice using splenocyte preparations and found that oleandrin increased the portion of both CD4^+^ and CD8^+^ T cells in the spleen. These data suggested that oleandrin treatment induced ICD and stimulated DC-mediated immune responses in vivo.

ICD activates anti-tumor immune response mainly by releasing DAMPs. CRT exposure, ATP secretion, and HMGB1 release are all indispensable for ICD, meaning that the absence of any one of these ICD hallmarks abolishes its efficacy^42^. In the present study, CRT exposure was observed in MCF7 and MDA-MB-231 breast cancer cells 6 h after oleandrin treatment. Consistent with the previous study, CRT exposure is earlier than cell apoptosis, which indicates CRT exposure was not the consequent event of DNA damage^9^. ER, where CRT locates, plays a crucial role in the maintenance of intracellular signal transduction, protein synthesis, and calcium homeostasis^43^. The RNA sequencing results suggested that oleandrin-induced ER stress was associated with ICD. Previous studies have shown that certain chemotherapy agents such as anthracyclines and oxaliplatin induce ER stress via activating the caspase-8 signaling pathway that regulates CRT exposure^25,44^. Caspase-8 hydrolyzes ER binding protein BAP31, which releases CRT from ER to cell surface^24,25^. However, in this study, Z-VAD-FMK, a caspase inhibitor, significantly inhibited the cell apoptosis, but had no significant effect on CRT exposure, indicating that the CRT exposure induced by oleandrin was not dependent on caspase pathway. Western blotting results further confirmed that oleandrin treatment activated ATF3 and CHOP via PERK-elF2α-ATF4 and IRE1-XBP1 pathways, but not ATF6. Pharmacological and genetic inhibition of PERK showed strong inhibitory effects on CHOP expression and CRT exposure, while IRE1 inhibition weakly suppressed oleandrin-induced ICD. Therefore, oleandrin induced ER stress mainly through the PERK axis and ER stress culminated in the translocation of the CRT to the cell surface, thereby generating an “eat-me” signal for DCs.

In the present study, ATP secretion was detectable after 6 h but HMGB1 release was confirmed after 24 h following oleandrin treatment. In the early stage of ER stress, in order to maintain cell homeostasis, cells activate autophagy which facilitates the release of ATP from dying cells^45,46^. ATP, as a “find-me” signal, recruits DCs to the dying cells. Distinct from ATP secretion, HMGB1 was passively released into the extracellular space at a later post-apoptotic time point, allowing HMGB1 to bind Toll-like receptor 4 on DCs and thus stimulate their antigen presentation functions^47^. Moreover, previous study demonstrated that CHOP regulates the release of HMGB1^48^. Therefore, we speculated CRT exposure, ATP secretion, and HMGB1 release were associated with ER stress (sFig. 3).

Immunotherapies represented by immune checkpoint inhibitors have shown promising therapeutic outcomes. PD-L1 overexpression, tumor mutation burden (TMB), and TILs affect the efficiency of immunosuppressive agents^49^. The number and function of TILs, especially CD4^+^ T cells, CD8^+^ T cells, and DCs, are key to the effectiveness of the immunotherapy^4^. Immune checkpoint inhibitors combining with ICD-inducing agents might be a more effective approach, especially in the treatment of tumors lacking immune cell invasion that are referred as “cold tumor”. Recently, it was demonstrated that immune checkpoint inhibitors increased the anti-tumor response while combined with ICD-inducing chemotherapy agents or radiation therapy^50,51^. Several clinical trials that combine checkpoint inhibitors with ICD inducers are ongoing, and the hope is that these combinations will increase the number of patients who can benefit from checkpoint inhibitor therapies. Further study is needed to demonstrate that whether oleandrin combined with immune checkpoint inhibitors will improve the efficacy of immunotherapy and reduce the side effects of chemotherapeutics through dose reduction.

## Conclusions

Oleandrin triggered ER stress and induced ICD-mediated immune destruction of breast cancer cells. Oleandrin combined with immune checkpoint inhibitors might improve the efficacy of immunotherapy.

## Supplementary information

Supplementary figure legends-clean.

sFig. 1.

sFig. 2.

sFig. 3.

## Data Availability

The datasets used and/or analyzed during the current study are available from the corresponding author on reasonable request.
