# Supplementary material for: Oleandrin, a cardiac glycoside, induces immunogenic cell death via the PERK/elF2α/ATF4/CHOP pathway in breast cancer
Source: Cell Death Dis. 2021 Mar 24;12(4):314. doi: 10.1038/s41419-021-03605-y (PMC7990929; doi:10.1038/s41419-021-03605-y)
Supplement: Supplementary file 1 — Supplementary figure legends-clean. [file 41419_2021_3605_MOESM1_ESM.docx]

**Title**

**Oleandrin, a cardiac glycoside, induces** **immunogenic cell death via the PERK/elF2α/ATF4/CHOP pathway in breast cancer**

**Running Title:** Oleandrin triggers anti-tumor immune activation

Xiaoxi Li^1^, Jian Zheng^2^, Shi Chen^1^, Fan-dong Meng^4^, Jing Ning^3^, Shu-lan Sun^1^

1 Central Laboratory, Cancer Hospital of China Medical University, Liaoning Cancer Hospital & Institute, Shenyang, Liaoning 110042, P. R. China.

2 Department of Thoracic Cancer, Cancer Hospital of China Medical University, Liaoning Cancer Hospital & Institute, Shenyang, Liaoning 110042, P. R. China.

3 Department of general medicine (VIP ward) & Department of tumor supportive and palliative medicine, Cancer Hospital of China Medical University，Liaoning Cancer Hospital & Institute, Shenyang, Liaoning 110042, P. R. China.

4 Molecular Oncology Laboratory of Cancer Research Institute, The First Affiliated Hospital of China Medical University, Shenyang, Liaoning, PR China.

**Correspondence:**

Shu-lan Sun: Central laboratory, Cancer Hospital of China Medical University, Liaoning Cancer Hospital & Institute, No.44 Xiaoheyan Road, Dadong District, Shenyang, Liaoning 110042, P. R. China. E-mail: sunshulan@cancerhosp-ln-cmu.com

**Supplementary figure legends**

sFig.1

MCF7, MDA-MB-231 cells **(A)** and PBMCs from volunteers **(B)** were stained with HLA-A2 and detected by flow cytometry. **(C)** PBMCs were cultured under IL-4, GM-CSF induction for 5 days. Morphology of the cells were observed. The scale bar of first and second pictures were 100 μm, and the third one was 50 μm. **(D)** After 5-day induction, cells were stained with CD11c. **(E)** DCs co-cultured with MDA-MB-231 cells were stained with CD45 and separated by cell sorter. **(F)** MCF7 and MDA-MB-231 cells were treated with oleandrin, and the expression of ATF6 were detected by western blotting.

sFig.2

**(A and B)** Splenocytes were collected from the tumor-bearing mice and detected by flow cytometry. The frequency of T cells and DCs were analyzed by staining with CD4, CD8, CD3, CD11b and CD11c. The results were represented as percentage of gated lymphocytes. n = 5, ***p < 0.01* vs. control. CON, control. **(C and D)** MCF7 and MDA-MB-231 cells were transfected with si-PERK or si-IRE1 for 48 h and then treated with oleandrin for 6 h. **(C)** The expressions of PERK, p-PERK, IRE1 and p-IRE1 were detected by Western blotting. β-actin was used as loading control. **(D)** Cells were stained with CRT and PI before detected by flow cytometry. The CRT-positive and PI-negative cells were showed in representative dot plots and quantification data. ***p <0.01.* ole, oleandrin.

sFig.3 Proposed model of oleandrin induced ICD in breast cancer

Oleandrin, as a Na^+^/K^+^ ATPase inhibitor, increases the concentration of Na^+^ and activated Na^+^/Ca^2+^ ion exchange channel on cell surface, which causes the influx of Ca^2+^. Loss of cellular homeostasis and disruption of Ca^2+^ leads to activation of ER stress associated pathways including PERK-elF2α-ATF4 and IRE1-XBP1. ER stress enhances the releasement of ATP and HMGB1. Moreover, ER stress induces CRT exposure to the cell surface. The release of these DAMPs signals eventually leads to the enhancement of immune response.
