# Supplementary figures and images for: Oleandrin, a cardiac glycoside, induces immunogenic cell death via the PERK/elF2α/ATF4/CHOP pathway in breast cancer
Source: Cell Death Dis. 2021 Mar 24;12(4):314. doi: 10.1038/s41419-021-03605-y (PMC7990929; doi:10.1038/s41419-021-03605-y)

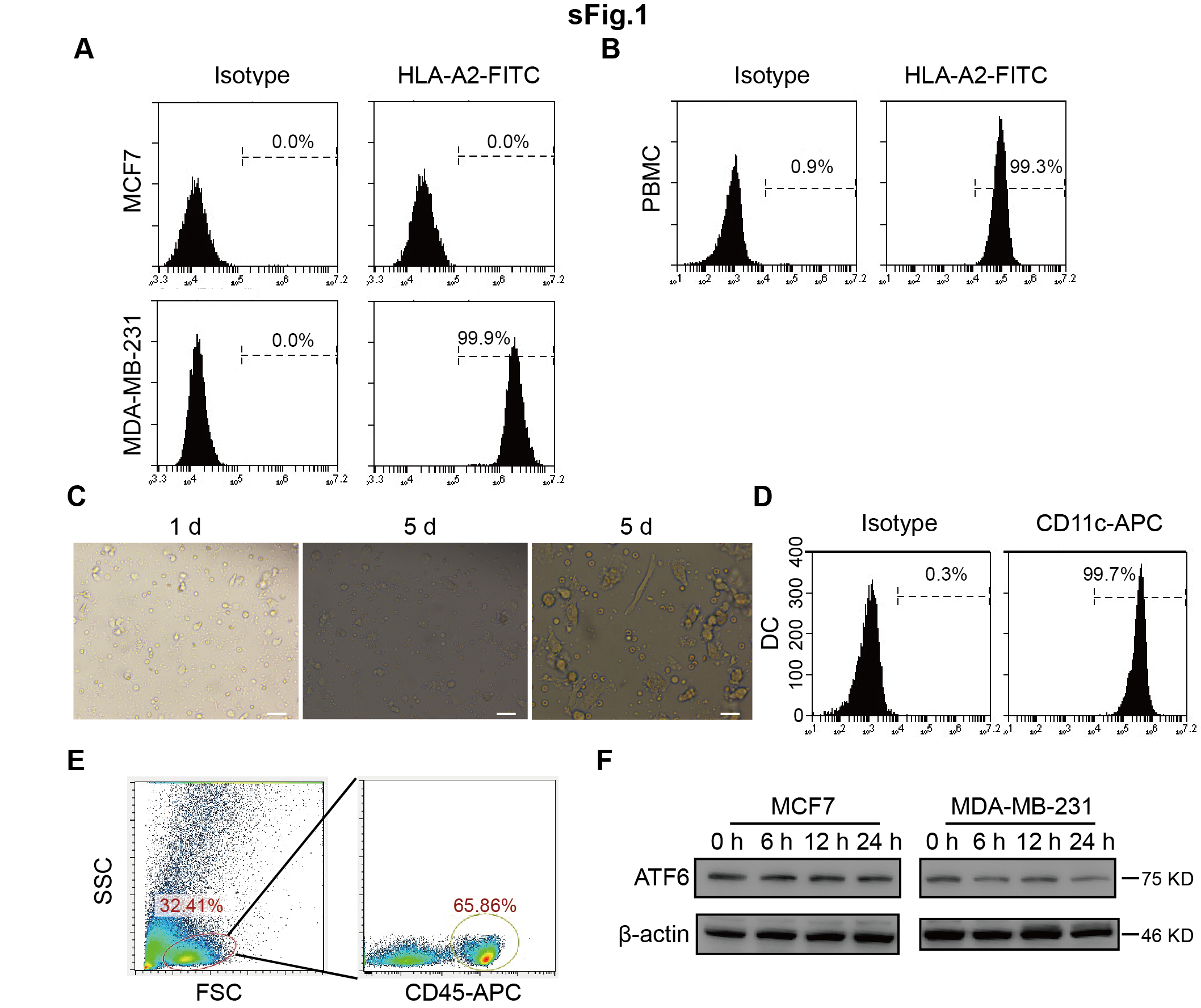

Supplement: Supplementary file 2 — sFig. 1. [file 41419_2021_3605_MOESM2_ESM.png]

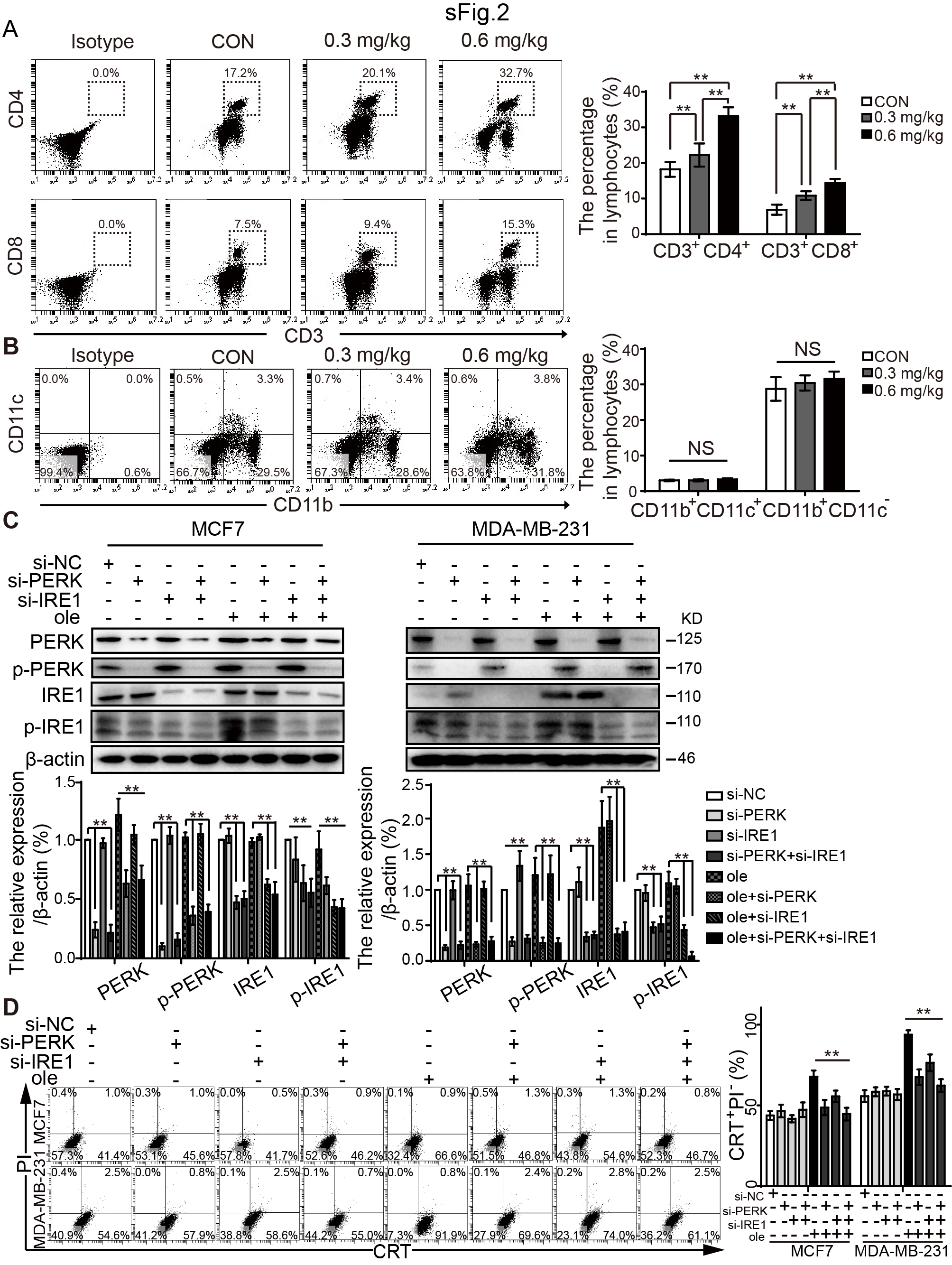

Supplement: Supplementary file 3 — sFig. 2. [file 41419_2021_3605_MOESM3_ESM.png]

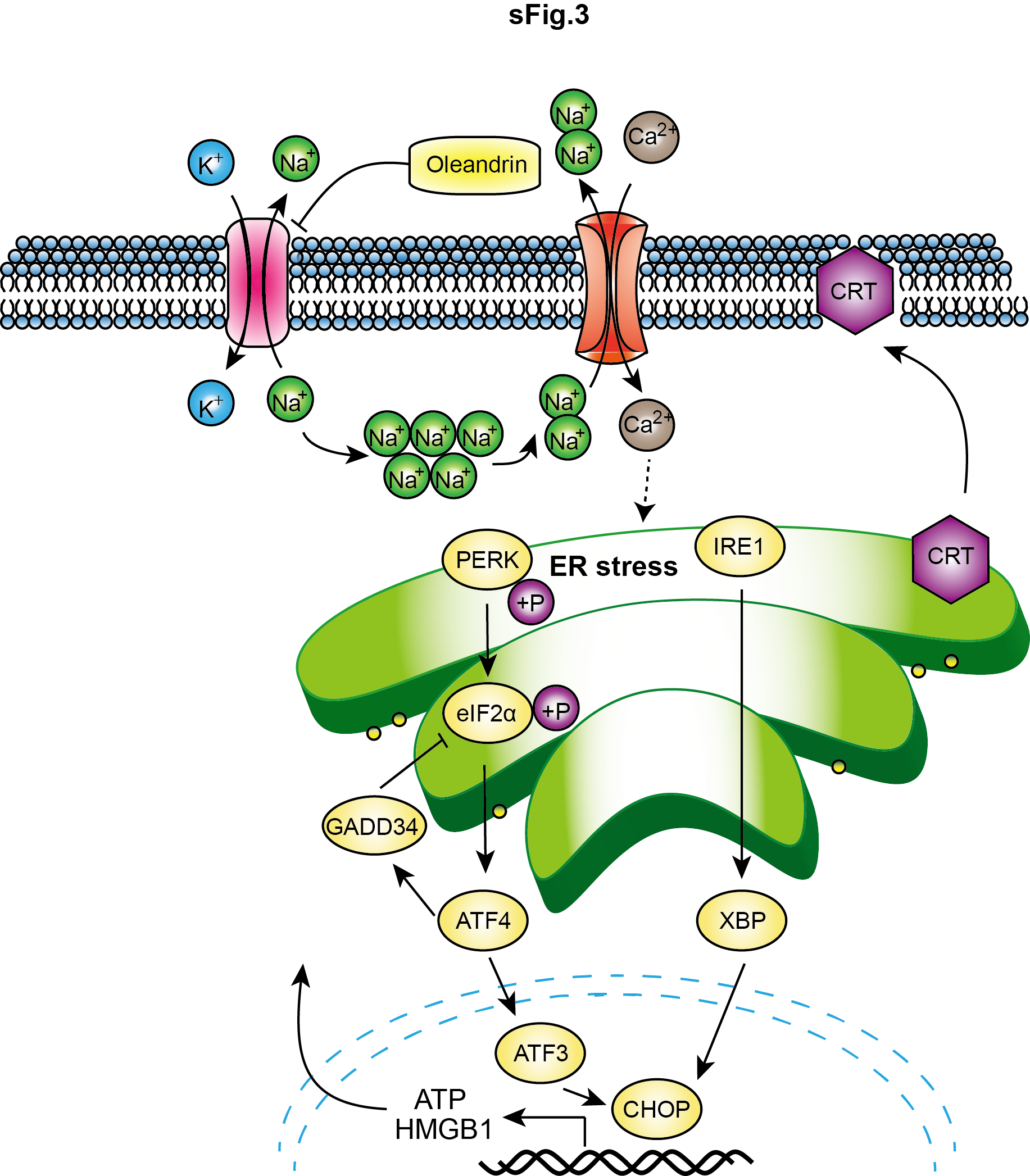

Supplement: Supplementary file 4 — sFig. 3. [file 41419_2021_3605_MOESM4_ESM.png]
